# Best practices for identifying men who have sex with men for corrections-based pre-exposure prophylaxis provision

**DOI:** 10.1186/s40352-019-0088-7

**Published:** 2019-04-13

**Authors:** Lauren Brinkley-Rubinstein, Meghan Peterson, Nickolas D. Zaller, David A. Wohl

**Affiliations:** 1 Department of Social Medicine, Center for Health Equity, University of North Carolina at Chapel Hill, 333 S. Columbia St., 341B MacNider Hall, Chapel Hill, NC 27599 USA; 2 Center for Health Equity Research, University of North Carolina at Chapel Hill, Chapel Hill, NC USA; 3 School of Public Health, Brown University, Providence, USA; 4 College of Public Health, University of Arkansas for Medical Sciences, Little Rock, AR USA; 5 Division of Infectious Diseases, School of Medicine, University of North Carolina at Chapel Hill, Chapel Hill, NC USA

**Keywords:** Incarceration, HIV, Pre-exposure prophylaxis, Qualitative research

## Abstract

**Purpose:**

Men who have sex with men (MSM) who are incarcerated are at increased risk for HIV acquisition, yet there are challenges associated with disclosing sexual identity/orientation among people who are incarcerated.

**Methods:**

The current study used semi-structured, qualitative interviews to explore attitudes and awareness of pre-exposure prophylaxis (PrEP) among 26 MSM who were incarcerated at the Rhode Island Department of Corrections.

**Results:**

Participants noted variable levels of willingness to disclose sexual identity/orientation.

**Conclusions:**

CJ institutions should consider involving medical staff and outside agencies when using the CDC PrEP guidelines or consider a WHO-based, rather than behavior-based, approach to determining candidacy for PrEP.

Men who have sex with men (MSM) are at increased risk for criminal justice (CJ) involvement and acquiring HIV (Lim et al., 2011; Beyrer et al., 2012) The incarceration rate of sexual minorities is three times that of the general population (Meyer et al., 2017). Almost 10% of men who are incarcerated report a prior same-sex experience (Meyer et al., 2017), and MSM who are incarcerated are two times more likely to perceive rape as a threat and three times more likely to request mental health services compared to heterosexual men who are incarcerated (Ratkalkar & Atkin-Plunk, 2017). However, many incarcerated MSM cite institutional distrust; and fear of bodily harm, violence, and social exclusion as barriers to disclosure of sexual orientation or identity (Peterson et al., 2018).

Pre-exposure prophylaxis (PrEP) is a HIV prevention strategy that is effective (Grant et al., 2010) among MSM and could be deployed in correctional settings (Brinkley-Rubinstein et al., 2018a). However, identifying MSM in jails and prisons is challenging given the concerns associated with disclosure of sexual behaviors (Ratkalkar & Atkin-Plunk, 2017; Peterson et al., 2018). While some correctional systems screen for sexual identity or orientation upon intake, many MSM likely do not disclose (Ratkalkar & Atkin-Plunk, 2017; Peterson et al., 2018).

Both the Centers for Disease Control and Prevention (CDC) and the World Health Organization (WHO) have recommendations for use of PrEP. The CDC recommends PrEP for HIV-negative individuals who report being gay, bisexual, or a MSM who has had condomless sex, has had a sexually transmitted infection in the past six months, or is in a serodiscordant relationship (CDC, 2014). The WHO has conceptualized PrEP eligibility differently and suggests that anyone belonging to a population that has an increased burden of HIV (defined as having a HIV incidence equal to or higher than 3 per 100 person-years) is at substantive risk, and, therefore, eligible for PrEP (World Health Organization, 2015). CJ populations have a prevalence of HIV that is three times that of the general population making individuals who are in the CJ system at substantive risk, regardless of risk behaviors (Maruschak, 2012).

Our research objective was to explore perceptions surrounding disclosure of sexual orientation and to identify candidates for PrEP in a correctional system. We provide considerations based on interviews with 26 incarcerated MSM in Rhode Island that may be instructive when designing and implementing PrEP programs in correctional settings (see Brinkley-Rubinstein, et al. for more information on the results of the overall study) (Brinkley-Rubinstein et al., 2018b). Specifically, we outline 1) four categories that characterize incarcerated MSM’s sexual orientation/identity disclosure practices; 2) suggestions for optimizing PrEP eligibility screening based on CDC guidelines; and 3) consideration of the adoption of screening based on the WHO’s definition of substantive risk.

## Methods

The current study was conducted at the Rhode Island Department of Corrections (RIDOC) in Cranston, Rhode Island. The RIDOC is a statewide prison and jail that houses all incarcerated individuals in the state. Approximately 15,000 men cycle through the RIDOC each year (Rhode Island Department of Corrections, 2011), and the prevalence of HIV is 3% (Rhode Island Department of Corrections, 2011).

Information related to sexual orientation/identity is collected during medical intake, which occurs in the first 48 h after incarceration. We received a waiver of documentation of consent, so participants only gave *verbal* consent before the interview. Inclusion criteria for this study included self-report of: being gay, bisexual or a man who has sex with men; ≥ 18; and being able to speak English. Interviews lasted 45–60 min and were conducted by three trained qualitative researchers. Participants were asked questions related to HIV risk, PrEP knowledge and interest, barriers to PrEP uptake and adherence, and experience disclosing sexual orientation/identity. All participants were asked open-ended questions about what might be the best way to identify MSM who would be candidates for PrEP. Interviews were conducted in a private room without correctional officers (COs) present. Interviews were digitally recorded and transcribed. All participants received $30 that was deposited into their commissary account. The study was approved by the institutional review board at the Miriam Hospital and RIDOC.

A general inductive approach was used to analyze data. Data were formulated into themes and categories in line with the research questions and objectives (Thomas, 2006). Two coders read through transcriptions looking for recurrent themes and patterns. Subsequently, each theme was given a code, and codes were compiled in a codebook. Quality checks were conducted on 20% of all transcripts for thematic agreement. Discrepancies in interpretation were resolved before final coding commenced.

## Results

A total of 26 incarcerated MSM at the RIDOC were interviewed. Sixteen were White, 8 were Black, and 2 were Hispanic. Participants ranged in age from 23 to 57 and the average age was 38. Across all interviews, individuals discussed perceptions on disclosure of sexual identity/orientation as well as preferences for PrEP provision.

### Four categories of disclosure

We found that incarcerated MSM fell into four disclosure categories: 1) those who wanted to disclose their sexual orientation/identity and were not concerned about others finding out; 2) those who were willing to disclose but only to certain individuals; 3) those who did not want to disclose their sexual orientation/identity, but felt they had no choice. These men expressed sentiments such as “everyone was going to find out no matter what” indicating a lack of agency and confidentiality in correctional settings; and 4) those who would not disclose (and were, therefore, not a part of our study). Many MSM who are incarcerated do not disclose sexual orientation/identity based on perceived stigma and fear of mistreatment (Ratkalkar & Atkin-Plunk, 2017; Peterson et al., 2018).

### Optimization of screening based on CDC guidelines

MSM who were open to disclosing their sexual orientation/identity suggested that if a correctional institution were to screen for PrEP eligibility using CDC’s guidelines, medical settings and partnerships with community-based organizations should be utilized. Participants perceived medical settings within correctional institutions as spaces for safe disclosure and emphasized that they trusted medical staff. One participant explained that he was more likely to trust medical staff because they did not have disciplinary powers: “I think that a lot of inmates would probably be more comfortable with a member of medical staff as opposed to somebody who’s involved on the floor or would be over you, such as a correctional officer or a lieutenant.” Another participant described his intake experience: “It was kind of comfortable in the little nurse’s office at intake cause it was just a nurse sitting here and a correctional officer. So you felt safe because the correctional officers are not going to say anything and the medical personnel aren’t going to say anything so it felt safe there to say what I needed to say and tell them what I was feeling and I needed some help.”

Participants explained that they would prefer external, community-based groups to offer PrEP services. Several participants explained that they would prefer PrEP be tied in with other services. Others stressed that they felt more comfortable in a one-on-one session with someone not employed by the correctional system. One participant noted: “It’s always good for somebody outside to come in because it means that there are people out there that care about your health. In here everybody thinks like oh they *have* to do this or they *have* to do that. If they [people who are incarcerated] see people come in from the outside to help them, that’s comfort and that shows that there’s people that care. [Like] he came this far to bring it to my attention, so why not [try it].”

### Consideration of screening based on the WHO’s definition of substantive risk

Many MSM endorsed an approach that was aligned with the WHO PrEP recommendations. This was particularly the screening preference among MSM who did not want to disclose their sexual orientation/identity but did so because they felt they had no choice, and, we speculate, that this would also be the preference among those who will not disclose in correctional settings. Participants hypothesized that the reasons why MSM choose not to disclose include: fear of mistreatment, distrust of medical and correctional institutions and stigma. Participants emphasized that one way to reach these MSM could be to offer PrEP in a way that was not explicitly tied to risk based on sexual orientation. One participant stated, “So maybe just being like hey it doesn’t matter what you do, and then making the drug and the information available.” Another participant felt that offering PrEP only to MSM functioned as a way of “othering” (Johnson et al., 2004) and explained: “To make that your demographic is discriminatory. It should be everyone.”

## Discussion

Incarcerated MSM expressed varying levels of comfort with disclosing sexual orientation/identity. For those with a greater willingness to disclose to correctional staff, the CDC approach to PrEP eligibility would be appropriate; however, there was a preference for sexual risk and PrEP to be discussed only with medical staff or individuals who were not affiliated with the correctional system the CDC approach to PrEP screening would be problematic for MSM who are reluctant to disclose sexual orientation/identity during incarceration, and, we suspect, for those not represented here because they will not disclose. For these MSM, the PrEP screening procedures based on the WHO definition of substantive risk would provide greater access.

Furthermore, participants discussed how attempts to provide PrEP to people who are incarcerated could utilize external organizations to foster trust, as MSM may be more likely to disclose risk to people who are not directly employed by correctional systems. This highlights how PrEP provision could use community partnerships and, subsequently, comprehensive discharge planning alongside external groups to best reach communities at risk.

The present study has some limitations. First, the RIDOC is a unique unified jail and prison system that may not necessarily represent other correctional facilities. Additionally, a larger proportion of people who are incarcerated in Rhode Island are White, which may not be generalizable to other communities. However, these findings are meant to provide a snapshot of lived experiences of incarcerated MSM in Rhode Island. Additionally, given that individuals who did not disclose sexual orientation/identity due to fear of stigma were not included in the study, the results could be biased.

The CDC estimates that over one 800,000 MSM are candidates for PrEP. There are calls for greater PrEP use among those at risk of HIV infection, including among more diverse populations than have accessed PrEP to date. Given their risks, individuals in the CJ system should be included in this expansion of PrEP. Our findings suggest that screening for PrEP be conducted in a manner that acknowledges the perceived and real threats that accompany sexual orientation disclosure. CJ institutions should consider involving medical staff and outside agencies when using the CDC PrEP guidelines or consider a WHO-based, rather than behavior-based, approach to determining candidacy for PrEP.
